# Amyloid and Tau Prediction of Cognitive and Functional Decline in Unimpaired Older Individuals: Longitudinal Data from the A4 and LEARN Studies

**DOI:** 10.14283/jpad.2024.122

**Published:** 2024-07-24

**Authors:** Reisa A. Sperling, M.C. Donohue, R.A. Rissman, K.A. Johnson, D.M. Rentz, J.D. Grill, J.L. Heidebrink, C. Jenkins, G. Jimenez-Maggiora, O. Langford, A. Liu, R. Raman, R. Yaari, K.C. Holdridge, J.R. Sims, P.S. Aisen, A4 and LEARN Study Teams

**Affiliations:** 1 Center for Alzheimer Research and Treatment, Department of Neurology, Brigham and Women's Hospital, Massachusetts General Hospital, Harvard Medical School, 60 Fenwood Road, 02115, Boston, MA, USA; 2 Alzheimer Therapeutic Research Institute, Keck School of Medicine, University of Southern California, San Diego, CA, USA; 3 Departments of Radiology and Neurology, Massachusetts General Hospital, Harvard Medical School, Boston, MA, USA; 4 Institute for Memory Impairments and Neurological Disorders, University of California, Irvine, CA, USA; 5 Department of Neurology and Michigan Alzheimer's Disease Center, University of Michigan, Ann Arbor, MI, USA; 6 Eli Lilly and Co, Indianapolis, IN, USA

**Keywords:** Amyloid, tau, imaging, biomarkers, cognitive decline

## Abstract

**Background:**

Converging evidence suggests that markers of Alzheimer's disease (AD) pathology in cognitively unimpaired older individuals are associated with high risk of cognitive decline and progression to functional impairment. The Anti-Amyloid Treatment in Asymptomatic Alzheimer's disease (A4) and Longitudinal Evaluation of Amyloid and Neurodegeneration Risk (LEARN) Studies enrolled a large cohort of cognitively normal older individuals across a range of baseline amyloid PET levels. Recent advances in AD blood-based biomarkers further enable the comparison of baseline markers in the prediction of longitudinal clinical outcomes.

**Objectives:**

We sought to evaluate whether biomarker indicators of higher levels of AD pathology at baseline predicted greater cognitive and functional decline, and to compare the relative predictive power of amyloid PET imaging, tau PET imaging, and a plasma P-tau217 assay.

**Design:**

All participants underwent baseline amyloid PET scan, plasma P-tau217; longitudinal cognitive testing with the Primary Alzheimer Cognitive Composite (PACC) every 6 months; and annual functional assessments with the clinical dementia rating (CDR), cognitive functional index (CFI), and activities of daily living (ADL) scales. Baseline tau PET scans were obtained in a subset of participants. Participants with elevated amyloid (Aβ+) on screening PET who met inclusion/exclusion criteria were randomized to receive placebo or solanezumab in a double-blind phase of the A4 Study over 240+ weeks. Participants who did not have elevated amyloid (Aβ−) but were otherwise eligible for the A4 Study were referred to the companion observational LEARN Study with the same outcome assessments over 240+ weeks.

**Setting:**

The A4 and LEARN Studies were conducted at 67 clinical trial sites in the United States, Canada, Japan and Australia.

**Participants:**

Older participants (ages 65–85) who were cognitively unimpaired at baseline (CDR-GS=0, MMSE 25–30 with educational adjustment, and Logical Memory scores within the normal range LMIIa 6–18) were eligible to continue in screening. Aβ+ participants were randomized to either placebo (n=583) or solanezumab (n=564) in the A4 Study. A subset of Aβ+ underwent tau PET imaging in A4 (n=350). Aβ− were enrolled into the LEARN Study (n=553).

**Measurements:**

Baseline 18-F Florbetapir amyloid PET, 18-F Flortaucipir tau PET in a subset and plasma P-tau217 with an electrochemiluminescence (ECL) immunoassay were evaluated as predictors of cognitive (PACC), and functional (CDR, CFI and ADL) change. Models were evaluated to explore the impact of baseline tertiles of amyloid PET and tertiles of plasma P-tau217 on cognitive and functional outcomes in the A4 Study compared to LEARN. Multivariable models were used to evaluate the unique and common variance explained in longitudinal outcomes based on baseline predictors, including effects for age, gender, education, race/ethnic group, APOEε4 carrier status, baseline PACC performance and treatment assignment in A4 participants (solanezumab vs placebo).

**Results:**

Higher baseline amyloid PET CL and P-tau217 levels were associated with faster rates of PACC decline, and increased likelihood of progression to functional impairment (CDR 0.5 or higher on two consecutive measurements), both across LEARN Aβ− and A4 Aβ+ (solanezumab and placebo arms). In analyses considering all baseline predictor variables, P-tau217 was the strongest predictor of PACC decline. Among participants in the highest tertiles of amyloid PET or P-tau217, >50% progressed to CDR 0.5 or greater. In the tau PET substudy, neocortical tau was the strongest predictor of PACC decline, but plasma P-tau217 contributed additional independent predictive variance in commonality variance models.

**Conclusions:**

In a large cohort of cognitively unimpaired individuals enrolled in a Phase 3 clinical trial and companion observational study, these findings confirm that higher baseline levels of amyloid and tau markers are associated with increased rates of cognitive decline and progression to functional impairment. Interestingly, plasma P-tau217 was the best predictor of decline in the overall sample, superior to baseline amyloid PET. Neocortical tau was the strongest predictor of cognitive decline in the subgroup with tau PET, suggesting that tau deposition is most closely linked to clinical decline. These findings indicate that biomarkers of AD pathology are useful to predict decline in an older asymptomatic population and may prove valuable in the selection of individuals for disease-modifying treatments.

## Introduction

The ability to accurately predict risk of cognitive and functional decline prior to symptom onset in Alzheimer's disease (AD) is critical to the identification of individuals who might benefit from very early intervention. Converging evidence from previous observational studies in cognitively unimpaired older individuals suggests that abnormal positron emission tomography (PET) imaging and fluid biomarkers of AD pathology are associated with increased risk of cognitive decline and progression to mild cognitive impairment and dementia (1, 2, 3).

The Anti-Amyloid Treatment in Asymptomatic Alzheimer's disease (A4) Study (4) was a Phase 3 clinical trial testing whether solanezumab, a monoclonal antibody against monomeric forms of amyloid-beta, could slow cognitive decline at the stage of asymptomatic or “preclinical” AD (5). Eligible participants were cognitively and functionally unimpaired with evidence of elevated amyloid on screening PET imaging. The Longitudinal Evaluation of Amyloid and Neurodegeneration Risk (LEARN) Study was launched as a companion observational study in a subset of cognitively unimpaired individuals who screen-failed for A4 on the basis of not showing elevated amyloid on screening PET. While the A4 Study did not demonstrate any evidence of clinical benefit of solanezumab treatment or lowering of amyloid PET below baseline levels (4), the A4 Study provides a rich dataset to explore cognitive and functional outcomes in a large, well-characterized cohort of biomarker positive, cognitively unimpaired older individuals (see Jimenez-Maggiora et al. in this special issue).

Overall, the elevated amyloid or “amyloid positive” (Aβ +) participants in both A4 treatment arms demonstrated cognitive and functional decline during the 4.5-year double-blind phase, in comparison to the amyloid “negative” (Aβ−) LEARN participants, who remained cognitively stable over the same interval. To follow-up on this initial observation, we sought to evaluate whether the level of amyloid on baseline PET imaging was predictive of longitudinal change in cognitive and functional outcomes during the A4 and LEARN Studies.

Since the launch of the A4 Study in 2014, which relied on amyloid PET to determine trial eligibility, there has been remarkable progress in blood-based biomarkers to detect evidence of AD pathology (6). In particular, plasma assays of phosphorylated tau at residue 217 (P-tau217) have emerged as sensitive and reliable indicators of early elevations in brain amyloid (7). More recently, P-tau217 has shown promise in prediction of cognitive decline in observational cohorts (8, 9, 10, 11). Banked plasma from baseline and selected longitudinal visits in both A4 and LEARN enabled measurement of baseline and longitudinal change in P-tau217 using a plasma immunoassay (see Rissman R et al in this special issue).

Tau PET imaging became available soon after the launch of the A4 Study and was incorporated into A4 as a substudy at a subset of sites. Tau PET was developed to measure forms of phosphorylated tau aggregated into paired helical filaments in tangles and neurites (12). Tau deposition measured by PET has been hypothesized to be more closely associated with cognitive impairment than amyloid pathology (13, 14).

In this study, we sought to evaluate whether baseline levels of AD pathology measured by molecular imaging and blood biomarkers were predictive of cognitive and functional outcomes over the 4.5-year doubleblind phase of A4 and the corresponding time period in the LEARN Study. We assessed models of variance explained to compare the relative utility of amyloid PET, tau PET, and plasma P-tau217 in predicting cognitive decline, including baseline demographics, cognitive and functional measures, and treatment assignment in the model. We assessed these variables across the LEARN and A4 cohorts, with the full range from Aβ− to Aβ+, and across the A4 Study Aβ+ treatment arms to determine whether higher levels of AD pathology at baseline predicted greater cognitive and functional decline in preclinical AD.

## Methods

The details of the methodology for A4 and LEARN screening and outcome measures have been previously described (4, 15) and are briefly summarized here. Participants eligible to screen for the A4 Study were age 65–85 and cognitively unimpaired with CDR-Global score=0, MMSE 25–30, and Weschler Memory Scale-Revised Logical Memory IIa Delayed Recall score of 6–18 (corresponding to approximately 1 SD above and below published age mean scores). All participants were in overall good health, and stable hypertension, diabetes, hypercholesterolemia, mild-moderate small vessel ischemic disease and other common medical ailments were permitted. All participants were required to have a study partner who was familiar with their cognitive function and daily activities.

Eligible participants underwent amyloid PET imaging with 18F-Florbetapir PET, measured with a mean cortical imaging standardized uptake value ratio (SUVr) using a cerebellar reference as previously described (16). Amyloid status for eligibility for A4 vs. LEARN (elevated or not elevated) was assessed using an algorithm combining quantitative SUVr methods and qualitative visual read performed at a central laboratory. SUVr's were converted to the standard Centiloid (CL) scale (17). Amyloid status was disclosed to all participants during screening with ongoing assessment and monitoring of psychological wellbeing by the A4 Ethics committee (18).

Participants with elevated amyloid on screening amyloid PET and who met all inclusion and exclusion criteria (see https://www.clinicaltrials.gov/study/NCT02008357) were randomly assigned in a 1:1 ratio to receive intravenous solanezumab or placebo. Randomization was stratified according to apolipoprotein E (APOE) e4 carrier status, education level (12 years or less or over 12 years) and trial site. Dosing was initially 400mg intravenously every four weeks but increased to 1600mg IV every four weeks later in the trial.

Participants who were otherwise eligible for A4 but did not show elevated amyloid were eligible to enroll in the LEARN Study (https://www.clinicaltrials.gov/study/NCT02488720) until the target enrollment was reached. The LEARN Study launched a year after the A4 Study began recruiting and was conducted at a subset of A4 sites. The LEARN participants underwent the same cognitive and functional assessments as the A4 Study participants.

### Outcome Assessments

The primary outcome in the A4 and LEARN Studies was the four-component Preclinical Alzheimer's Cognitive Composite Scale (PACC) (19), consisting of the Free Recall plus Total (sum of free and cued) score from the Free and Cued Selective Reminding Test (FCSRT, range 0–96) (20), the delayed paragraph recall on the Logical Memory IIa test from the Wechsler Memory Scale and alternative paragraphs developed by Jeri Morris et al. (range 0–25) (21, 22), the Digit-Symbol Substitution Test from the Wechsler Adult Intelligence scale-Revised (23), and the Mini Mental Status Examination (MMSE) (range 0–30) (24). Three versions of the component subtests were alternated to minimize practice effects, and testing was conducted by trained site psychometrists blinded to treatment arm assignment and adverse events. Each component score was converted to a z-score by subtracting the baseline mean for that component and dividing by the baseline standard deviation for that component, resulting in the sum of four z-scores, with negative scores indicating cognitive worsening (25).

Secondary endpoints included the Cognitive Function Index (CFI) (26), a set of 15 questions that capture subjective concerns related to cognitive function administered to the participant and study partner separately and then summed (range 0–30, see Amariglio et al in this Issue); the ADCS-Activities of Daily Living Prevention Instrument (ADL) assessed by the study partner (range 0 to 45 (27)); Clinical Dementia Rating – Global Score (CDR-G >0 indicating mild cognitive impairment or dementia); and Clinical Dementia Rating Sum of Boxes (CDR-SB) (range 0 to 18, with higher scores indicating greater global functioning impairment) ((28) see also Rentz et al. in this issue)

Baseline imaging and biomarker predictors included 18-F florbetapir amyloid PET (measured in average cortical composite CL as described above) and plasma P-tau217 (immunoassay, see Rissman R et al in this issue for methods). Tau PET imaging was acquired with 18-F flortaucipir in a subset of participants, with an average SUVr measured in a standard template composite of early necortical regions: inferior temporal, fusiform, middle temporal, inferior parietal cortices.

### Statistical Analyses

Natural cubic spline models were used to investigate the trajectories of the PACC, CFI, and ADL and CDR-SB scales (29). Models included effects for age, education, apolipoprotein E e4 (APOE4) carrier status, tertile group (by amyloid PET or P-tau217), and two spline basis expansion terms for time per tertile group. Clinical Dementia Rating Global (CDR-G) Score progression rates by amyloid PET and P-tau217 groups were estimated by Kaplan-Meier. CDR-GS Progression was defined as two consecutive CDR-GS above 0, or an endpoint CDR-GS above 0 at the end of the double-blind period.

A two-step procedure was used to assess the proportion of variance (R^2^) of PACC change explained by each predictor. First, linear mixed-effect models were fit to each group (placebo, solanezumab, and LEARN). These models included fixed effect for cognitive test alternate version and two spline basis expansion terms for time; and participant-specific random intercepts and slopes. Participant-specific estimated change from baseline at week 240 was derived from these models. In step two, the participant-specific change scores were submitted to a “commonality analysis” (30, 31) based on ordinary least squares regression models considering ten baseline predictors: Amyloid PET, P-tau217, Baseline PACC, CFI, age, APOE **e4** carrier status, female sex, education (years), Race-Ethnic Under-Represented Group (URG) membership, and treatment group (where applicable). Commonality analysis partitions the R^2^ for each predictor into R^2^ unique to each predictor and R^2^ shared with other predictors by fitting regression models with every combination of predictors.

Shaded regions in graphics represent 95% confidence intervals. All p-values and 95% CIs are nominal (i.e., not adjusted for multiplicity). All analyses were conducted using R version 4.4.0.

## Results

A total of 1718 participants who were cognitively unimpaired at baseline are included in these analyses (see Table 1), including Aβ+ participants who were randomized to either placebo (n=583) or solanezumab (n=564) in A4, and Aβ− participants (n=553) in LEARN (see Table 1 for subset with P-tau217 measures available). Aβ+ participants randomized in the A4 Study were slightly older, had higher rates of APOEε4 carriers, lower MMSE scores at baseline and, by selection, showed greater baseline amyloid PET CL values, compared to LEARN participants. The baseline mean amyloid PET CL was 66CL for A4 and 4CL for LEARN. Baseline P-tau217 was a good predictor of baseline amyloid PET, with an AUC of .89 predicting >33CL (based on the quantitative SUVr cutpoint of 1.15) across A4 and LEARN participants (see also Rissman R et al in this issue).

**Table 1:** Baseline characteristics for A4 and LEARN with Amyloid PET and P-tau217

	Solanezumab (N=521)	Placebo (N=535)	All A4 (N=1056)	LEARN (N=469)	p-value for A4 vs LEARN
Age — yr	72.0 (4.6)	71.9 (5.0)	71.9 (4.8)	70.5 (4.3)	< 0.001
Female Sex	297 (57.0%)	320 (59.8%)	617 (58.4%)	289 (61.6%)	0.241
Education — yr	16.6 (2.7)	16.5 (2.9)	16.5 (2.8)	16.7 (2.6)	0.256
Racial categories					0.045
American Indian or Alaskan Native	1 (0.2%)	0 (0.0%)	1 (0.1%)	5 (1.1%)	
Asian	9 (1.7%)	13 (2.4%)	22 (2.1%)	9 (1.9%)	
Black or African American	10 (1.9%)	12 (2.2%)	22 (2.1%)	10 (2.1%)	
More than one race	5 (1.0%)	3 (0.6%)	8 (0.8%)	5 (1.1%)	
Unknown or Not Reported	4 (0.8%)	3 (0.6%)	7 (0.7%)	0 (0.0%)	
White	492 (94.4%)	504 (94.2%)	996 (94.3%)	440 (93.8%)	
Ethnicity					0.929
Hispanic or Latino	14 (2.7%)	18 (3.4%)	32 (3.0%)	15 (3.2%)	
Not Hispanic or Latino	501 (96.2%)	512 (95.7%)	1013 (95.9%)	450 (95.9%)	
Unknown or Not reported	6 (1.2%)	5 (0.9%)	11 (1.0%)	4 (0.9%)	
Family history of dementia (parent or sibling)	381 (73.1%)	411 (76.8%)	792 (75.0%)	308 (65.7%)	< 0.001
APOE Genotype					< 0.001
*ε*2/*ε*2	1 (0.2%)	0 (0.0%)	1 (0.1%)	4 (0.9%)	
*ε*2/*ε*3	27 (5.2%)	30 (5.6%)	57 (5.4%)	58 (12.4%)	
*ε*2*ε*e4	12 (2.3%)	20 (3.7%)	32 (3.0%)	8 (1.7%)	
*ε*3/*ε*3	181 (34.7%)	189 (35.3%)	370 (35.0%)	303 (64.6%)	
*ε*3/*ε*4	257 (49.3%)	252 (47.1%)	509 (48.2%)	94 (20.0%)	
*ε*4/*ε*4	43 (8.3%)	44 (8.2%)	87 (8.2%)	2 (0.4%)	
P-tau217	0.3 (0.2)	0.3 (0.1)	0.3 (0.2)	0.2 (0.1)	< 0.001
FBP SUVr	1.3 (0.2)	1.3 (0.2)	1.3 (0.2)	1.0 (0.1)	< 0.001
FBP Centiloid	66.5 (33.1)	65.8 (32.3)	66.2 (32.7)	4.7 (12.4)	< 0.001
PACC	0.0 (2.7)	0.0 (2.7)	0.0 (2.7)	0.9 (2.3)	< 0.001
LM Delayed Recall	12.7 (3.9)	12.7 (3.5)	12.7 (3.7)	13.7 (3.3)	< 0.001
MMSE	28.8 (1.3)	28.8 (1.3)	28.8 (1.3)	29.0 (1.2)	< 0.001
CFI Combined	2.4 (2.2)	2.3 (2.1)	2.3 (2.2)	1.8 (1.9)	< 0.001
ADL Partner	43.4 (2.6)	43.5 (2.6)	43.5 (2.6)	43.9 (1.9)	< 0.001
CDR-SB	0.1 (0.2)	0.0 (0.1)	0.1 (0.2)	0.0 (0.1)	0.083


Values are means (SD) or counts (%). P-values for testing differences between A4 and LEARN derived from two-sample t-tests or Pearson's chi-squared tests. APOE denotes apolipoprotein E, FBP is 18-F Florbetapir Amyloid PET in Standard Uptake Value Ratio (SUVr) and Centiloid, PACC is Preclinical Alzheimer Cognitive Composite, LM is Weschler Logical Memory Delayed Recall, MMSE is Mini-Mental Status Examination, CFI is Cognitive Function Index Combined Participant and Study Partner, ADL is Activities of Daily Living Study Partner, and CDR-SB is Clinical Dementia Rating Scale-Sum of Boxes.

We first evaluated amyloid PET and P-tau217 as predictors of PACC decline across the LEARN and the full A4 population. As we did not observe a significant treatment effect on PACC decline, we combined the A4 arms but did include treatment assignment as a covariate in later model analyses.

**Figure 1:**
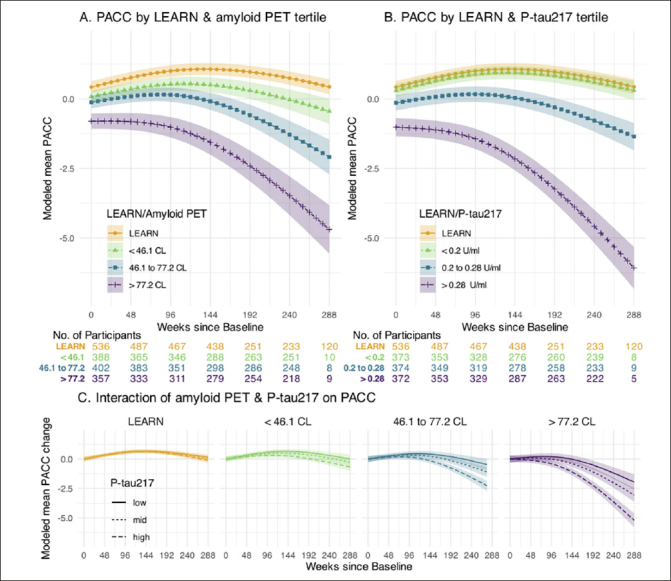
Cognitive Change by Amyloid PET and Plasma P-tau217 levels Panel A depicts modeled mean Preclinical Alzheimer Cognitive Composite (PACC) for those in LEARN and each tertile of baseline amyloid PET in A4. Panel B depicts modeled mean PACC for those in LEARN and each tertile of baseline P-tau 217 using a similar approach. Panel C depicts the interaction of baseline amyloid PET and P-tau 217 levels. Each of the four panels of Panel C shows the modeled mean PACC change according to the tertiles of baseline P-tau217 within the indicated amyloid PET group. All models assumes a natural cubic spline for time with two degrees of freedom for per group and control for age, APOEε4 carrier status, education and PACC version; and all models assume heterogeneous Toeplitz variance-covariance. Shaded regions are 95% confidence intervals.

Figure 1 illustrates the PACC trajectories comparing LEARN to the tertiles of amyloid PET (Figure 1A) and plasma P-tau217 (Figure 1B) in the A4 cohort. The Aβ− LEARN cohort demonstrated evidence of an early practice effect on the PACC and did not decline below their baseline at the 240-week timepoint. In A4, the lowest tertile of amyloid (<46CL) demonstrated minimal decline, performing slightly worse on the PACC compared to LEARN participants by 240 weeks. The middle tertile (46.1 to 77.2 CL) demonstrated decline in the PACC at the later timepoints, whereas the highest tertile of amyloid PET CL (>77CL) showed worse PACC performance at baseline and showed the greatest PACC decline over the 240 weeks. A similar pattern was observed across the P-tau217 tertiles. LEARN and the lowest P-tau217 tertile in A4 were overlapping in PACC change, and the highest A4 P-tau217 tertile clearly demonstrated the steepest PACC decline (Figure 1C).

**Figure 2:**
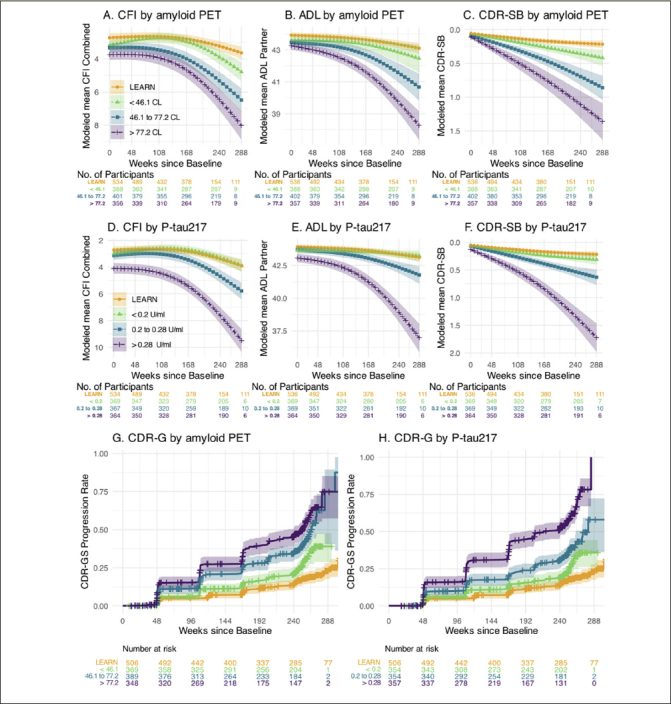
Functional Outcomes by amyloid PET and Plasma P-tau217 The Cognitive Function Index (CFI) combined participant and study partner trajectories for LEARN and A4 tertiles of baseline amyloid PET in Panel A and for plasma P-tau 217 in Panel D. Activities of Daily Living Prevention Instrument (ADL) study partner trajectories are shown by amyloid PET (Panel B) and plasma P-tau217 (Panel E). The Clinical Dementia Rating Sum of Boxes (CDR-SB) trajectories are shown by amyloid PET (Panel C) and P-tau217 (Panel F). Kaplan-Meier estimated Clinical Dementia Rating Global Score (CDR-GS) progression rates are shown by amyloid PET (Panel G) and P-tau 217 (Panel H). CDR-GS Progression is defined as two consecutive CDR-GS above 0, or final endpoint CDR-GS above 0. Shaded regions are 95% confidence intervals.

Prediction of Decline on Functional Outcomes: We performed similar analyses predicting change on the pseudo-continuous measures of functional and clinical progression: the CFI (participant+study partner combined) CFI, ADL (study partner), and CDR-SB measure. Figure 2 demonstrates the similar patterns observed across tertiles of amyloid PET and P-tau217 in each of these outcome measures. We also utilized time to event analyses to evaluate progression to CDR-GS of 0.5 or higher (requiring two consecutive CDR-GS>0 except at final time point). The highest tertile of P-tau217 showed the greatest risk of progression to functional impairment, exceeding 50% progression to CDR-GS of 0.5 or higher by the end of the 240 weeks (Figure 2H).

**Figure 3:**
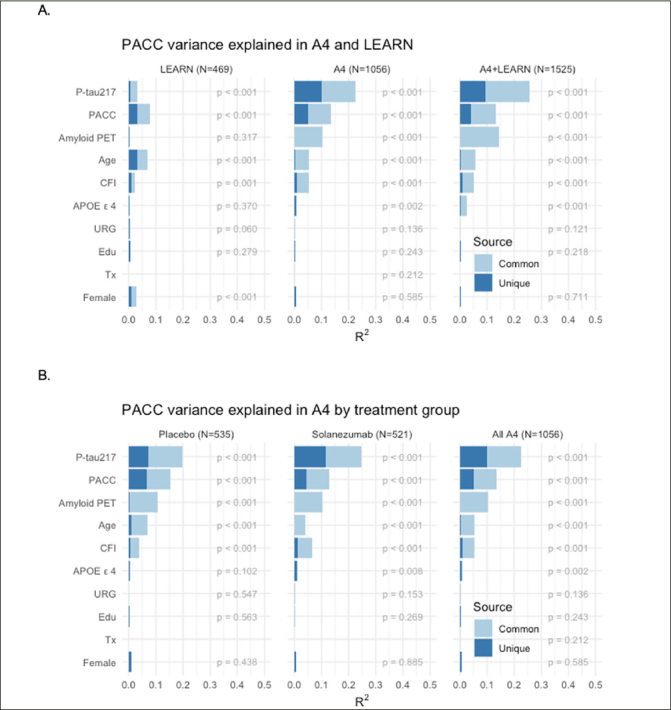
PACC Change Variance Explained Full Sample Across LEARN and A4 and within A4 Treatment Groups PACC change variance explained (R^2^) in LEARN and A4 (A: Top Row) and within and across A4 treatment arms (B: Bottom Row) by each baseline predictor, partitioned into common and unique (R^2^). Participant-specific summaries of PACC change from baseline at week 240 were estimated from linear mixed-effects models two spline basis expansion terms for time and adjusting for PACC test version and participant-specific random intercepts and slopes. These change scores were then submitted to ordinary least squares regression with each of the indicated baseline predictors. The R^2^ from these models are indicated by the sum of light and dark blue bars, and the nominal p-value for each predictor is shown.

Models of Unique and Common Variance Explained in Cognitive Change: We performed a series of models to determine the relative contribution of each baseline variable in predicting PACC decline, in the sample who had data from all baseline measures available. Not surprisingly, across the full LEARN and A4 cohorts, amyloid PET and P-tau217 markers explained the majority of variance in PACC decline (Figure 3A). In the LEARN cohort, baseline PACC performance and age explained the most variance in decline, with much less, but nominally significant, variance explained by baseline amyloid PET and P-tau217, even in this Aβ- group. The total variance (R^2^) explained by all predictors in PACC change was: LEARN: R^2^=0.163; LEARN+All A4: R^2^=0.341.

We then evaluated these baseline predictors in just the Aβ+ participants across the two A4 treatment arms (Figure 3B). P-tau217 consistently made the largest unique contribution to PACC decline R^2^. In models including all baseline variables, P-tau217 continued to be the strongest predictor. Interestingly, amyloid PET had very little unique contribution to R^2^, suggesting that P-tau217 was capturing the amyloid PET-related predictive capability. Baseline performance on the PACC and CFI remained nominally significant predictors, as did age, APOE ε4 carrier status, gender, and education. We did not observe any significant predictive value in PACC change conferred by race/ethnicity or treatment with solanezumab. The total variance explained by these predictor variables in PACC decline in the full A4 cohort was: placebo arm: R^2^=0.324; solanezumab arm R^2^=0.340; combined A4 arms R^2^=0.329.

**Figure 4:**
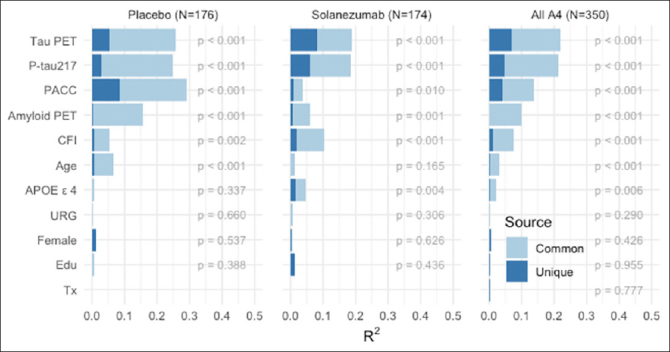
A4 Tau PET Substudy PACC Change Variance Explained PACC change variance explained (R^2^) in A4 Tau PET Substudy (N-350) by each baseline predictor considered partitioned into common and unique (R^2^), within and across A4 treatment arms. Participant-specific summaries of PACC change from baseline at week 240 are estimated from linear mixed-effects models two spline basis expansion terms for time and adjusting for PACC test version and participant-specific random intercepts and slopes. These change scores were then submitted to ordinary least squares regression with each of the indicated baseline predictors. The R^2^ from these models are indicated by the sum of light and dark blue bars, and the nominal p-value for the predictor is shown.

Finally, we repeated these analyses in the A4 tau PET substudy (N=350 participants with all baseline variables available; demographics in Supplemental Table 1). Only a very small number of LEARN participants had tau PET and P-tau217 values available, so we did not include LEARN data in these analyses. Across A4 treatment arms, both P-tau217 and neocortical tau PET composite showed a similar proportion of explained variance in predicting PACC decline (Figure 4). Neocortical tau contributed the largest R^2^, but P-tau217 remained a significant predictor, suggesting that these measures are capturing unique sources of variance in explaining cognitive decline. Baseline amyloid PET, had very little unique R^2^, suggesting the predictive value of amyloid PET is captured fully in the other variables. The total variance explained in the tau PET substudy was: placebo arm: R^2^=0.515; solanezumab arm R^2^=0.356; combined A4 arms R^2^=0.401.

## Discussion

Our findings demonstrate that higher levels of amyloid and tau biomarkers are associated with greater cognitive and functional decline over 4.5 years in a group of older individuals who were cognitively and functionally unimpaired at baseline. The observed cognitive and functional decline in the Aβ+ A4 cohort, particularly among those in the highest tertile of amyloid PET and plasma P-tau217, supports the view that cognitively unimpaired individuals with evidence of brain amyloidosis represent a very early stage of AD, specifically “preclinical AD” rather than being at “increased risk for AD” (32).

Plasma P-tau217 was the strongest predictor of cognitive decline within the overall A4 cohort (across both treatment arms), and across LEARN and the A4 placebo arm analyses, with a smaller contribution within the LEARN cohort alone. The predictive value of baseline P-tau217 on cognitive decline was greater than baseline amyloid PET. Although P-tau217 is thought to primarily reflect amyloid plaque burden (33), these findings suggest that P-tau217 levels may reflect the degree to which amyloid is triggering downstream change, including tauopathy (i.e., “the amyloid is bothering the tau”). P-tau217 is thought to reflect an early stage of tau phosphorylation related to misfolding of tau into the paired helical filaments that form tangles, as well as tau neurites that surround amyloid plaques. The predictive value of plasma P-tau217, in comparison to amyloid PET, has important implications for improving the efficiency of early intervention and primary prevention trial designs. These findings provide additional support for the supposition that plasma P-tau217 may prove useful as a clinical tool for selecting candidates for amyloid-lowering immunotherapy (34).

Although P-tau217 is associated with elevated amyloid and tau PET levels, there have been several reports that plasma and CSF P-tau217 levels begin to rise prior to standard thresholds used to determine amyloid PET positivity in autosomal dominant AD (35). Interestingly in the LEARN cohort, which had very low amyloid PET levels at baseline (mean of 4CL), P-tau217 was still a significant predictor of cognitive trajectories. This may point to a pathophysiologic role of amyloid and tau in individuals below the A4 cutoff for amyloid elevation, supporting future intervention studies at an even earlier stage in the disease spectrum. Furthermore, even within the group of just Aβ+ participants enrolled in A4, higher P-tau217 levels predicted more rapid PACC decline, suggesting that plasma measures may have predictive value across the full range of amyloid accumulation observed in early stages of AD.

Although the A4 and LEARN Studies enrolled participants within a very narrow range of “normal” cognitive performance, baseline PACC remained a significant predictor of decline even with the biomarker and imaging variables in the model, suggesting that there are likely other factors that may reflect cognitive resilience or vulnerability in baseline performance. This finding may also reflect baseline differences in PACC related to time since the accumulation of amyloid and tau pathology began (36), as we observed differences in cognitive and functional measures related to amyloid status at screening (15). We also note that baseline PACC performance was a strong predictor in LEARN. LEARN participants did not demonstrate cognitive decline over the nearly 5 year period, instead showing evidence of practice effects early in the study and finishing at the baseline level of performance overall. This finding suggests that in the absence of substantial amyloid accumulation in brain, age-related cognitive decline is not observed over a 5 year period.

These findings also indicate that the degree of AD pathology is a strong predictor of likelihood of functional decline. In the highest tertile of baseline amyloid PET and plasma P-tau217 values, nearly 50% of the participants progressed to functional impairment, defined as two consecutive global CDR 0.5 or higher ratings or at their final CDR timepoint in the study. This result suggests that cognitively unimpaired individuals with most elevated AD biomarkers are indeed likely to progress to symptomatic AD within 5 years, in contrast to some of the epidemiological projections that employed only binary biomarker categorization for life-time risk of impairment (37).

The level of tau PET signal in early neocortical regions was the strongest predictor of cognitive decline in the subset of the A4 Study who underwent flortaucipir tau PET imaging, consistent with the hypothesis that tau spreading into the neocortex signals impending cognitive impairment (13). Neocortical tau PET level was more predictive of cognitive decline than plasma P-tau217 levels among cognitively unimpaired older individuals, consistent with previous reports in patients with symptomatic AD (38). Interestingly, P-tau217 still contributed additional unique variance in prediction of cognitive change. Previous work has suggested that plasma P-tau217 may predict future tau deposition observable on tau PET imaging (39), and may indicate earlier stages of the pathophysiological process that leads to AD tauopathy. Further work is ongoing to explore the specific anatomic progression of tauopathy in the A4 Study participants, including exploring early tau deposition in medial temporal lobe regions (40), and the anatomic-temporal relationship with cognitive decline.

Consistent with the lack of benefit observed in the solanezumab group compared to placebo reported in the primary analyses (4), we did not observe any significant effect of treatment group in these analyses including amyloid and tau markers. Nevertheless, these findings inform the design and interpretation of ongoing and future trials. The substantial progression observed on the CDR, CFI and ADL scales, measures of clinically meaningful change, among A4 participants with higher levels of amyloid and tau pathology, compared to the minimal decline seen in LEARN support the feasibility of establishing clinical efficacy of candidate therapeutics in the preclinical AD population. Baseline amyloid PET burden and level of P-tau217 was a strong predictor of subsequent decline, consistent with a pivotal role for amyloid accumulation in driving the pathophysiological process of AD and associated impairment. These findings suggest that aggressive amyloid reduction may be necessary even at the preclinical stage of AD to prevent cognitive and functional decline, and support the rationale for testing amyloid-removing antibodies in preclinical AD populations, including secondary prevention trials such as the AHEAD Study testing lecanemab (NCT04468659)(41) and TRAILBLAZER-ALZ 3 testing donanemab (NCT05026866).

### Limitations

These participants (both A4 and LEARN) volunteered and met basic screening criteria for a clinical trial aiming to prevent cognitive decline in preclinical AD, and thus may not represent a generalizable population. These participants were in general good health, willing to commit to multiple visits over several years, and had higher rates of family history of dementia than the general older population (15). Despite substantial efforts to improve the racial and ethnic diversity in these studies, the A4 and LEARN cohorts were not adequately representative of all older individuals at risk for cognitive decline. Recent work has highlighted potential differences in the prevalence of abnormal amyloid PET and AD biomarkers among underrepresented race and ethnic groups (URG) (42, 43, 44). We did not observe any differences in rates of cognitive decline comparing participants from URG to overrepresented white participants, in analyses including other demographic factors, baseline performance, and baseline biomarkers and imaging variables, but are very limited in our ability to draw conclusions with the inadequate race/ethnic diversity in the A4 and LEARN cohorts.

Both A4 and LEARN studies experienced prolonged disruption of activities at the sites because of social distancing measures due to the COVID-19 pandemic. Some LEARN sites were closed for observational study activities for over one year, and there was greater attrition in the LEARN Study than A4. It should also be acknowledged that the participants in LEARN and A4 were aware of their amyloid PET status (only disclosed as elevated vs. non-elevated (18), which could have influenced their assessments. However, the binary disclosure at baseline is less likely to account for the increasing differences in decline over the 4.5-year period and could not account for the observed association of greater amyloid load with more rapid decline among the Aβ+ A4 arms. The P-tau217 results were not disclosed at any point during the studies.

Finally, with these amyloid and tau baseline variables, we explained only 30–52% of the total variance in PACC decline across the various models and cohorts, suggesting that additional measures may be useful in more fully capturing the contributing factors. We chose to include only one variable from each of the categories (baseline amyloid PET, plasma, tau PET, cognition, function) to predict cognitive decline to avoid overfitting the models but will continue to explore additional variables from these modalities. Ongoing work with vascular risk factors, such as systolic blood pressure, and magnetic resonance imaging (MRI) measures, including hippocampal volume, white matter hyperintensities, functional connectivity (see Boyle et al in this issue), and microhemorrhage measures (see Shirzadi et al in this issue), may add to the ability to predict cognitive and functional decline. Recent work suggests that vascular risk may be synergistic with amyloid in accelerating tau and cognitive decline (45) and that imaging markers of vascular health may contribute to resilience (46). Furthermore, neuropathological studies clearly suggest that other proteinopathies, such as TDP-43 and alphasynuclein, likely contribute to cognitive decline in this age range (47, 48), but are currently lacking quantitative biomarkers that are easily obtained in vivo. Despite all these complexities, our findings suggest that a simple plasma assay for P-tau217 may be very useful for prediction of future cognitive decline in asymptomatic individuals, and to identify those most likely to benefit from very early intervention with anti-amyloid therapeutics.

## Electronic Supplementary Material

Supplementary material, approximately 16.0 KB.

*Funding:* The A4 and LEARN Studies were supported by a public-private-philanthropic partnership which included funding from the National Institute of Aging of the National Institutes of Health (R01 AG063689, U19AG010483 and U24AG057437), Eli Lilly (also the supplier of active medication and placebo), the Alzheimer's Association, the Accelerating Medicines Partnership through the Foundation for the National Institutes of Health, the GHR Foundation, the Davis Alzheimer Prevention Program, the Yugilbar Foundation, an anonymous foundation, and additional private donors to Brigham and Women's Hospital, with in-kind support from Avid Radiopharmaceuticals, Cogstate, Albert Einstein College of Medicine and the Foundation for Neurologic Diseases.

*Ethical Standards:* Approval from an institutional review board or ethics committee was obtained at each of the sites. All participants and their study partners provided written informed consent prior to data collection, which included consent for data sharing.

*Disclosure of Potential Conflicts of Interest:* RAS reports grant support from the National Institutes on Aging, National Institutes of Health, Alzheimer's Association, GHR Foundation, and Gates Ventures. She has received trial research funding from Eisai and Eli Lilly for public-private partnership trials. She reported serving as a consultant for AbbVie, AC Immune, Alector, Biohaven, Bristol-Myers-Squibb, Ionis, Janssen, Genentech, Merck, Prothena, Roche, and Vaxxinity. MCD has received research funding from the National Institutes of Health, Janssen, Eli Lilly, and Eisai, reports consulting fees from Roche and his spouse is a full-time employee of Janssen. KAJ has received research funding from National Institutes on Aging, National Institutes of Health, Alzheimer's Association, and the GHR Foundation. He has served as a consultant for Merck, Novartis, Janssen, and Prothena. RAR has research support from the National Institute on Aging, the Alzheimer's Association and is a consultant for Amydis Inc, Bioivt, Lexeo, Keystone Bio, Allyx, DiamiR, Ionis and PrecisionMed. CJ has received research support from the National Institutes of Health (NIH), the Alzheimer's Association, American Heart Association, Eli Lilly and Eisai. DMR received salary and research support from the National Institutes of Health and has received payment or honoraria from USC Institute on Methods and Protocols for Advancement of Clinical Trials in ADRD (IMPACT AD) course and External Advisory Boards from the University of California-Davis, Washington University, Boston University and Northwestern. She has also received travel support to ACTC meetings, to the University of California Advisory Board Meeting and the Washington University Advisory Board Meeting. JG has received research support from the National Institutes of Health (NIH), the Alzheimer's Association, BrightFocus Foundation, Eli Lilly, Biogen, Genentech, and Eisai. He has received personal fees for providing consulting to SiteRx and editorial service to Alzheimer's & Dementia. JLH has received research support from the National Institutes of Health, the Alzheimer's Association, Eli Lilly, and Eisai and has received honoraria from USC Institute on Methods and Protocols for Advancement of Clinical Trials in ADRD (IMPACT AD) RR has received research support from the National Institutes of Health (NIH), the Alzheimer's Association, American Heart Association, Eli Lilly and Eisai. AL has received research support from the National Institutes of Health (NIH), the Alzheimer's Association, American Heart Association, Eli Lilly and Eisai. OL has received research support from the National Institutes of Health (NIH), the Alzheimer's Association, American Heart Association, Eli Lilly and Eisai. GJM has received research support from the National Institutes of Health (NIH), the Alzheimer's Association, American Heart Association, Gates Ventures, Eli Lilly, and Eisai. RY is an employee and minor shareholder of Eli Lilly and Company. KCH is an employee and minor shareholder of Eli Lilly and Company. JS is an employee and minor shareholder of Eli Lilly and Company. PSA has received grants or contracts from the National Institutes of Health (NIH), Alzheimer's Association, Foundation for NIH (FNIH), Lilly, Janssen and Eisai and consulting fees from Merck, Biogen, AbbVie, Roche, and Immunobrain Checkpoint.

## References

[1] Donohue MC, Sperling RA, Petersen R, Sun CK, Weiner MW, Aisen PS (2017). Association Between Elevated Brain Amyloid and Subsequent Cognitive Decline Among Cognitively Normal Persons. JAMA.

[2] Ossenkoppele R, Smith R, Mattsson-Carlgren N, Groot C, Leuzy A, Strandberg O (2021). Accuracy of Tau Positron Emission Tomography as a Prognostic Marker in Preclinical and Prodromal Alzheimer Disease: A Head-to-Head Comparison Against Amyloid Positron Emission Tomography and Magnetic Resonance Imaging. JAMA Neurol.

[3] Jack CR, Wiste HJ, Algeciras-Schimnich A, Weigand SD, Figdore DJ, Lowe VJ (2024). Comparison of plasma biomarkers and amyloid PET for predicting memory decline in cognitively unimpaired individuals. Alzheimers Dement.

[4] Sperling RA, Donohue MC, Raman R, Rafii MS, Johnson K, Masters CL (2023). Trial of Solanezumab in Preclinical Alzheimer's Disease. N Engl J Med.

[5] Sperling RA, Aisen PS, Beckett LA, Bennett DA, Craft S, Fagan AM (2011). Toward defining the preclinical stages of Alzheimer's disease: Recommendations from the National Institute on Aging-Alzheimer's Association workgroups on diagnostic guidelines for Alzheimer's disease. Alzheimers Dement.

[6] Barthelemy NR, Salvado G, Schindler SE, He Y, Janelidze S, Collij LE (2024). Highly accurate blood test for Alzheimer's disease is similar or superior to clinical cerebrospinal fluid tests. Nat Med.

[7] Teunissen CE, Thijssen EH, Verberk IMW (2020). Plasma p-tau217: from ‘new kid' to most promising candidate for Alzheimer's disease blood test. Brain.

[8] Mattsson-Carlgren N, Janelidze S, Palmqvist S, Cullen N, Svenningsson AL, Strandberg O (2020). Longitudinal plasma p-tau217 is increased in early stages of Alzheimer's disease. Brain.

[9] Jonaitis EM, Janelidze S, Cody KA, Langhough R, Du L, Chin NA (2023). Plasma phosphorylated tau 217 in preclinical Alzheimer's disease. Brain Commun.

[10] Yakoub Y., Gonzalez-Ortiz F., Ashton N.J., Dery C., Strikwerda-Brown C., St-Onge F. (2024). Plasma p-tau217 predicts cognitive impairments up to ten years before onset in normal older adults. medRxiv.

[11] Niimi Y, Janelidze S, Sato K, Tomita N, Tsukamoto T, Kato T (2024). Combining plasma Abeta and p-tau217 improves detection of brain amyloid in non-demented elderly. Alzheimers Res Ther.

[12] Pontecorvo MJ, Keene CD, Beach TG, Montine TJ, Arora AK, Devous MD (2020). Comparison of regional flortaucipir PET with quantitative tau immunohistochemistry in three subjects with Alzheimer's disease pathology: a clinicopathological study. EJNMMI Res.

[13] Johnson KA, Schultz A, Betensky RA, Becker JA, Sepulcre J, Rentz D (2016). Tau positron emission tomographic imaging in aging and early Alzheimer disease. Ann Neurol.

[14] Ossenkoppele R, Pichet Binette A, Groot C, Smith R, Strandberg O, Palmqvist S (2022). Amyloid and tau PET-positive cognitively unimpaired individuals are at high risk for future cognitive decline. Nat Med.

[15] Sperling RA, Donohue MC, Raman R, Sun CK, Yaari R, Holdridge K (2020). Association of Factors With Elevated Amyloid Burden in Clinically Normal Older Individuals. JAMA Neurol.

[16] Royse SK, Minhas DS, Lopresti BJ, Murphy A, Ward T, Koeppe RA (2021). Validation of amyloid PET positivity thresholds in centiloids: a multisite PET study approach. Alzheimers Res Ther.

[17] Klunk WE, Koeppe RA, Price JC, Benzinger TL, Devous MD, Jagust WJ (2015). The Centiloid Project: standardizing quantitative amyloid plaque estimation by PET. Alzheimers Dement.

[18] Grill JD, Raman R, Ernstrom K, Sultzer DL, Burns JM, Donohue MC (2020). Short-term Psychological Outcomes of Disclosing Amyloid Imaging Results to Research Participants Who Do Not Have Cognitive Impairment. JAMA Neurol.

[19] Papp KV, Rofael H, Veroff AE, Donohue MC, Wang S, Randolph C (2022). Sensitivity of the Preclinical Alzheimer's Cognitive Composite (PACC), PACC5, and Repeatable Battery for Neuropsychological Status (RBANS) to Amyloid Status in Preclinical Alzheimer's Disease -Atabecestat Phase 2b/3 EARLY Clinical Trial. J Prev Alzheimers Dis.

[20] Grober E, Hall CB, Lipton RB, Zonderman AB, Resnick SM, Kawas C (2008). Memory impairment, executive dysfunction, and intellectual decline in preclinical Alzheimer's disease. J Int Neuropsychol Soc.

[21] Morris J, Swier-Vosnos A, Woodworth C, Umfleet LG, Czipri S, Kopald B (2014). Development of alternate paragraphs for the Logical Memory subtest of the Wechsler Memory Scale-IV. Appl Neuropsychol Adult.

[22] D W (1987). WMS-R: Wechsler Memory Scale-Revised: Manual.

[23] D W (1981). Wechsler Adult Intelligence Scale-Revised.

[24] Folstein MF, Folstein SE, McHugh PR (1975). “Mini-mental state”. A practical method for grading the cognitive state of patients for the clinician. J Psychiatr Res.

[25] Donohue MC, Sperling RA, Salmon DP, Rentz DM, Raman R, Thomas RG (2014). The preclinical Alzheimer cognitive composite: measuring amyloid-related decline. JAMA Neurol.

[26] Amariglio RE, Donohue MC, Marshall GA, Rentz DM, Salmon DP, Ferris SH (2015). Tracking early decline in cognitive function in older individuals at risk for Alzheimer disease dementia: the Alzheimer's Disease Cooperative Study Cognitive Function Instrument. JAMA Neurol.

[27] Galasko D, Bennett DA, Sano M, Marson D, Kaye J, Edland SD (2006). ADCS Prevention Instrument Project: assessment of instrumental activities of daily living for community-dwelling elderly individuals in dementia prevention clinical trials. Alzheimer Dis Assoc Disord.

[28] Morris JC (1993). The Clinical Dementia Rating (CDR): current version and scoring rules. Neurology.

[29] Donohue MC, Langford O, Insel PS, van Dyck CH, Petersen RC, Craft S (2023). Natural cubic splines for the analysis of Alzheimer's clinical trials. Pharm Stat.

[30] Siebold DR, McPhee RD (1979). Commonality analysis: A method for decomposing explained variance in multiple regression analyses. Human Communication Research.

[31] Nimon KF, Oswald FL (2013). Understanding the results of multiple linear regression: Beyond standardized regression coefficients. Organizational Research Methods.

[32] Jack CR, Bennett DA, Blennow K, Carrillo MC, Dunn B, Haeberlein SB (2018). NIA-AA Research Framework: Toward a biological definition of Alzheimer's disease. Alzheimers Dement.

[33] Janelidze S, Palmqvist S, Leuzy A, Stomrud E, Verberk IMW, Zetterberg H (2022). Detecting amyloid positivity in early Alzheimer's disease using combinations of plasma Abeta42/Abeta40 and p-tau. Alzheimers Dement.

[34] Mattsson-Carlgren N, Collij LE, Stomrud E, Pichet Binette A, Ossenkoppele R, Smith R (2024). Plasma Biomarker Strategy for Selecting Patients With Alzheimer Disease for Antiamyloid Immunotherapies. JAMA Neurol.

[35] Aguillon D, Langella S, Chen Y, Sanchez JS, Su Y, Vila-Castelar C (2023). Plasma p-tau217 predicts in vivo brain pathology and cognition in autosomal dominant Alzheimer's disease. Alzheimers Dement.

[36] Cody K.A., Langhough R.E., Zammit M.D., Clark L., Chin N., Christian B.T. (2024). Characterizing brain tau and cognitive decline along the amyloid timeline in Alzheimer's disease. Brain.

[37] Brookmeyer R, Abdalla N (2019). Multistate models and lifetime risk estimation: Application to Alzheimer's disease. Stat Med.

[38] Smith R, Cullen NC, Pichet Binette A, Leuzy A, Blennow K, Zetterberg H (2023). Tau-PET is superior to phospho-tau when predicting cognitive decline in symptomatic AD patients. Alzheimers Dement.

[39] Dore V, Doecke JD, Saad ZS, Triana-Baltzer G, Slemmon R, Krishnadas N (2022). Plasma p217+tau versus NAV4694 amyloid and MK6240 tau PET across the Alzheimer's continuum. Alzheimers Dement (Amst).

[40] Sanchez J.S., Becker J.A., Jacobs H.I.L., Hanseeuw B.J., Jiang S., Schultz A.P. (2021). The cortical origin and initial spread of medial temporal tauopathy in Alzheimer's disease assessed with positron emission tomography. Sci Transl Med.

[41] Rafii MS, Sperling RA, Donohue MC, Zhou J, Roberts C, Irizarry MC (2023). The AHEAD 3–45 Study: Design of a prevention trial for Alzheimer's disease. Alzheimers Dement.

[42] Deters KD, Napolioni V, Sperling RA, Greicius MD, Mayeux R, Hohman T (2021). Amyloid PET Imaging in Self-Identified Non-Hispanic Black Participants of the Anti-Amyloid in Asymptomatic Alzheimer's Disease (A4) Study. Neurology.

[43] Wilkins CH, Windon CC, Dilworth-Anderson P, Romanoff J, Gatsonis C, Hanna L (2022). Racial and Ethnic Differences in Amyloid PET Positivity in Individuals With Mild Cognitive Impairment or Dementia: A Secondary Analysis of the Imaging Dementia-Evidence for Amyloid Scanning (IDEAS) Cohort Study. JAMA Neurol.

[44] Molina-Henry D.P., Raman R., Liu A., Langford O., Johnson K., Shum L.K. (2024). Racial and ethnic differences in plasma biomarker eligibility for a preclinical Alzheimer's disease trial. Alzheimers Dement.

[45] Yau WW, Shirzadi Z, Yang HS, Ikoba AP, Rabin JS, Properzi MJ (2022). Tau Mediates Synergistic Influence of Vascular Risk and Abeta on Cognitive Decline. Ann Neurol.

[46] Qiu T, Liu ZQ, Rheault F, Legarreta JH, Valcourt Caron A, St-Onge F (2024). Structural white matter properties and cognitive resilience to tau pathology. Alzheimers Dement.

[47] Nelson PT, Dickson DW, Trojanowski JQ, Jack CR, Boyle PA, Arfanakis K (2019). Limbic-predominant age-related TDP-43 encephalopathy (LATE): consensus working group report. Brain.

[48] Boyle PA, Yu L, Leurgans SE, Wilson RS, Brookmeyer R, Schneider JA (2019). Attributable risk of Alzheimer's dementia attributed to age-related neuropathologies. Ann Neurol.

