# Supplementary material for: Amyloid and Tau Prediction of Cognitive and Functional Decline in Unimpaired Older Individuals: Longitudinal Data from the A4 and LEARN Studies
Source: J Prev Alzheimers Dis. 2024 Jul 24;11(4):802–13. doi: 10.14283/jpad.2024.122 (PMC11266444; doi:10.14283/jpad.2024.122)
Supplement: Supplementary file 1 — Supplementary material, approximately 16.0 KB. [file mmc1.docx]

**Supplemental Table 1: Demographics for Tau PET Substudy Sample**

## **Table S1: Baseline characteristics for A4 Tau PET and P-tau217**

|  | Solanezumab (N=174) | Placebo (N=176) | All A4 (N=350) |
| --- | --- | --- | --- |
| **Age -- y** | 72.5 (4.7) | 71.7 (4.9) | 72.1 (4.8) |
| **Female Sex** | 94 (54.0%) | 107 (60.8%) | 201 (57.4%) |
| **Education -- y** | 16.3 (2.8) | 16.0 (2.9) | 16.2 (2.8) |
| **Racial categories** |  |  |  |
| American Indian or Alaskan Native | 0 (0.0%) | 0 (0.0%) | 0 (0.0%) |
| Asian | 8 (4.6%) | 9 (5.1%) | 17 (4.9%) |
| Black or African American | 4 (2.3%) | 4 (2.3%) | 8 (2.3%) |
| More than one race | 3 (1.7%) | 1 (0.6%) | 4 (1.1%) |
| Native Hawaiian or Other Pacific Islander | 0 (0.0%) | 0 (0.0%) | 0 (0.0%) |
| Unknown or Not Reported | 1 (0.6%) | 1 (0.6%) | 2 (0.6%) |
| White | 158 (90.8%) | 161 (91.5%) | 319 (91.1%) |
| **Ethnicity** |  |  |  |
| Hispanic or Latino | 4 (2.3%) | 5 (2.8%) | 9 (2.6%) |
| Not Hispanic or Latino | 167 (96.0%) | 168 (95.5%) | 335 (95.7%) |
| Unknown or Not reported | 3 (1.7%) | 3 (1.7%) | 6 (1.7%) |
| **Family history of dementia (parent or sibling)** | 127 (73.0%) | 130 (73.9%) | 257 (73.4%) |
| **APOE Genotype** |  |  |  |
| ε2/ ε2 | 1 (0.6%) | 0 (0.0%) | 1 (0.3%) |
| ε2/ ε3 | 6 (3.4%) | 11 (6.2%) | 17 (4.9%) |
| ε2/ ε4 | 4 (2.3%) | 8 (4.5%) | 12 (3.4%) |
| ε3/ ε3 | 67 (38.5%) | 64 (36.4%) | 131 (37.4%) |
| ε3/ ε4 | 86 (49.4%) | 78 (44.3%) | 164 (46.9%) |
| ε4/ ε4 | 10 (5.7%) | 15 (8.5%) | 25 (7.1%) |
| **P-tau 217** | 0.3 (0.2) | 0.3 (0.1) | 0.3 (0.1) |
| **FBP SUVr** | 1.3 (0.2) | 1.3 (0.2) | 1.3 (0.2) |
| **FBP Centiloid** | 63.3 (32.3) | 63.2 (31.1) | 63.2 (31.7) |
| **PACC** | -0.1 (2.6) | -0.3 (2.9) | -0.2 (2.8) |
| **LM Delayed Recall** | 12.7 (4.0) | 12.5 (3.7) | 12.6 (3.8) |
| **MMSE** | 28.7 (1.3) | 28.6 (1.3) | 28.6 (1.3) |
| **CFI Combined** | 2.6 (2.4) | 2.2 (1.9) | 2.4 (2.2) |
| **ADL Partner** | 43.5 (2.3) | 43.5 (2.7) | 43.5 (2.5) |
| **CDR-SB** | 0.1 (0.2) | 0.1 (0.2) | 0.1 (0.2) |

Values are means (SD) or counts (%). APOE denotes apolipoprotein E, FBP is 18-F Florbetapir Amyloid PET in Standard Uptake Value Ratio (SUVr) and Ceniloid, PACC is Preclinical Alzheimer Cognitive Composite, LM is Weschler Logical Memory Delayed Recall, MMSE is Mini-Mental Status Examination, CFI is Cognitive Function Index Combined Self Participant and Study Partner, ADL is Activities of Daily Living Study Partner, and CDR-SB is Clinical Dementia Rating Scale-Sum of Boxes.
